# Abundant organic nitrogen enhances natamycin biosynthesis by increasing NAD(P) metabolic pathway activity in *Streptomyces gilvosporeus* F607

**DOI:** 10.3389/fmicb.2025.1684019

**Published:** 2025-10-27

**Authors:** Zhihui Meng, Minghui Yu, Jiahui Wang, Haijun Li, Wenhui Gao, Peitong Yang, Xingyu Cui, Peipei Zhang, Jiafang Fu, Guangxiang Cao, Gongli Zong

**Affiliations:** ^1^Biomedical Sciences College and Shandong Medicinal Biotechnology Centre, Key Lab for Genetic Engineering and Synthetic Biology of Shandong Province, Shandong First Medical University and Shandong Academy of Medical Sciences, Ji'nan, China; ^2^Shandong Freda Biotechnology Co., Ltd., Linyi, China

**Keywords:** natamycin biosynthesis, NAD (P) metabolism, NMN, NadD, metabolic engineering

## Abstract

Natamycin, a polyene macrolide antifungal produced by several Streptomyces species, is widely used as an industrial mold inhibitor in food preservation. Although nitrogen source effects on its production have been extensively studied, its underlying regulatory mechanisms remain unclear. Using transcriptome and metabolome analyses, we found that natamycin production increase by 2.8-fold during cultivation of *Streptomyces gilvosporeus* F607 in organic nitrogen-rich medium, accompanied by upregulation of 10 genes involved in NAD(P) biosynthesis and corresponding elevations in the levels of NAD or NADP with 1.81-fold and 1.04-fold increases. A novel multisubstrate enzyme, designated nicotinic acid mononucleotide (NaMN) adenylyltransferase (NadDsg), was demonstrated to slightly favor nicotinamide mononucleotide (NMN) over NaMN. Supplementation of *S. gilvosporeus* F607 with 10 mg/L NMN and overexpression of a site-directed mutant enzyme (NadDsg_RYKK_, at positions of R^17^Y^20^K^26^R^168^) promoted natamycin biosynthesis by 43.1%, increasing production from 4.67 g/L to 6.68 g/L. These findings indicate that an abundance of organic nitrogen primarily enhances natamycin biosynthesis by elevating NAD(P) levels, providing valuable insights for medium optimization and strain engineering toward high-yield natamycin production.

## 1 Introduction

Nitrogen sources, ranging from simple compounds (e.g., ammonium, nitrate, and nitrite) to complex organic forms, are crucial for primary and secondary metabolism in *Streptomyces* (Krysenko and Wohlleben, 2024). During growth, the diverse nitrogen sources are converted into amino acids and other nitrogen-containing compounds to support the synthesis of cellular building blocks (Fink et al., 2002). Primary metabolism provides nitrogen-containing intermediates or precursors for secondary metabolism (Ryu et al., 2006), which incorporates diverse compounds, such as ammonium, nitrate, amino acids, amino sugars, and urea (Rodríguez et al., 2018). Antibiotic production in *Streptomyces* is largely affected by the availability of nitrogen sources (Martín and Liras, 2010; Passmore et al., 2021).

Natamycin, a glycosylated 26-membered tetraene macrolide antifungal (Aparicio et al., 2000; Volpon and Lancelin, 2002), is produced by *Streptomyces* species including *S. natalensis* (Aparicio et al., 2000), *S. gilvosporeus* (Liang et al., 2008), *S. lydicus* (Wu et al., 2013), and *S. chattanoogensis* (Du et al., 2009). Its antifungal activity is dependent on hydrophobic interactions between the polyene of natamycin and sterols in the fungal cell wall (Antón et al., 2004; Aparicio et al., 2016; Yangla and Yldz, 2016). Natamycin has emerged as a major mold inhibitor in the food industry (Yangla and Yldz, 2016), and shows potential in preventing the proliferation of prostate cancer cells (Vasquez et al., 2020). Optimization of the cultivation medium for natamycin production in *S. natalensis* has been reported (Farid et al., 2020), and recent findings confirm that nitrogen metabolism plays a crucial role in enhancing its yield (Wang et al., 2025a,b). In *S. gilvosporeus* strain AG-2, pentose phosphate pathway enhancement, tricarboxylic acid cycle modulation, and overexpression of acetyl-CoA and malonyl-CoA via the nitrogen metabolism regulator *glnR* were shown to promote natamycin production (Wang et al., 2025b).

Reductive steps in most antibiotic biosynthetic pathways require the co-factor Nicotinamide Adenine Dinucleotide (NAD) Phosphate (NADPH), whose intracellular levels are reportedly influenced by the activities of glucose-6-phosphate dehydrogenase and 6-phosphogluconate dehydrogenase in the pentose phosphate pathway (PPP) (Butler et al., 2002). Natamycin, like other macrocyclic polyketides, is synthesized by type I modular Polyketide Synthases (PKSs), which sequentially assemble carbon chains from small acyl precursors (Aparicio et al., 2003). During synthesis of the polyketide macrolactone backbone, the ketosynthase domain of PKS catalyzes ketoreduction using the 4-*pro*-S hydride of NADPH, yielding a β-hydroxy thioester product (Passmore et al., 2021). Thus, biosynthesis of polyene macrolide polyketides requires adequate NADPH as a co-factor providing reducing power (Borgos et al., 2006; Zhang et al., 2020). Enhanced nitrogen metabolism has been proposed to indirectly increase NADPH reducing power by upregulating glycolysis and the PPP pathway (Wang et al., 2025b). However, the mechanism by which organic nitrogen sources affect natamycin biosynthesis remains unclear.

In this study, we investigated the effects of organic nitrogen on natamycin biosynthesis in *S. gilvosporeus* strain F607 through transcriptome and metabolome analyses. We found that increased biosynthesis of NAD (P), originating from the aspartate (Asp) oxidase and quinolinate (QA) synthesis pathways, enhanced reducing power during natamycin biosynthesis. Furthermore, modification of nicotinic acid mononucleotide adenylyltransferase (NadD), the rate-limiting enzyme in the NAD (P) synthesis pathway, reduced the accumulation of Nicotinic Acid (NA) and improved both NAD(P) levels and natamycin yield. Our findings reveal specific alterations in the NAD (P) synthesis pathway in *S. gilvosporeus* and provide insights into medium optimization and fermentation design for improving natamycin production.

## 2 Methods

### 2.1 Strains and cultures

*S. gilvosporeus* strain F607 (GenBank accession no. CP020569) was cultured in seed medium containing 10 g/L glucose, 5 g/L peptone, 3 g/L yeast extract, and 3 g/L malt extract for 24 h. To induce fermentation for natamycin production, 5% of the seed culture was used to inoculate low-nitrogen NTL medium (2.0 g/L soy peptone, 0.45 g/L yeast extract, 2.0 g/L NaCl, 1.0 g/L MgSO_4_, 60.0 g/L glucose, pH 7.5) or high-nitrogen NTH medium (20 g/L soy peptone, 4.5 g/L yeast extract, 2.0 g/L NaCl, 1.0 g/L MgSO_4_, 60.0 g/L glucose, pH 7.5). The strains used are listed in Supplementary Table 1.

### 2.2 Ultra-High Performance Liquid Chromatography (UHPLC) coupled with Q Exactive (QE) Mass Spectrometry (MS) detection of intracellular metabolites

A 4-mL bacterial culture grown in NTH fermentation broth was centrifuged at 10,000 rpm to collect the cells, which were then washed thrice with phosphate-buffered saline solution. 1,000 μL extract solution (methanol: acetonitrile: water = 2: 2: 1, with isotopically-labeled internal standard mixture) was added into 20 mg sample. Then the samples were homogenized at 35 Hz for 4 min and sonicated for 5 min in ice-water bath. The homogenization and sonication cycle were repeated for 3 times. Then the samples were incubated for 1 h at −40°C and centrifuged at 12,000 rpm for 15 min at 4°C. The resulting supernatant was transferred to a fresh glass vial for analysis. The quality control (QC) sample was prepared by mixing an equal aliquot of the supernatants from all of the samples. UHPLC-QE-MS detection of intracellular metabolites was performed by Biotree Biomedical Technology Co., Ltd. (Shanghai, China), using a Vanquish UHPLC system (Thermo Fisher Scientific, MA, USA) with a UPLC ethylene-bridged hybrid (BEH) amide column (2.1 × 100 mm, 1.7 μm) coupled to an Orbitrap QE HFX mass spectrometer (Thermo Fisher Scientific). Mobile phase A (25 mmol/L ammonium acetate and 25 mmol/L ammonia hydroxide in water [pH 9.75]) and mobile phase B (100% acetonitrile) were used for both positive and negative modes of electrospray ionization (ESI). The ESI source conditions were as follows: sheath gas flow rate, 30 Arb; auxiliary gas flow rate, 25 Arb; capillary temperature, 350° C; full MS resolution, 60,000; tandem MS/MS resolution, 7,500; collision energy, 10/30/60 in normalized collision energy mode; and spray voltage, 3.6 kV (positive) or 23.2 kV (negative). The raw data were converted to the mzXML format using ProteoWizard and processed with an in-house program, which was developed using R and based on XCMS, for peak detection, extraction, alignment, and integration. Then an in-house MS2 database (BiotreeDB) was applied in metabolite annotation. The cutoff for annotation was set at 0.3.

### 2.3 Transcriptomic analyses

For transcriptome sequencing and analysis, F607 mycelia were harvested after 24, 60, and 120 h of culture in different media. 1 mL fermentation culture was centrifuged at 5,000 rpm at 4 °C for 5 mins for twice and resuspend by TE buffer. Bacterial suspension was grinded in a clean, liquid nitrogen cooled mortar. Add liquid nitrogen once every 1 min, and grind 2-3 times. RNA was extracted by using the RNA Extraction Kit (Baiteke, Beijing). RNA integrity was assessed using the Bioanalyzer 2,100 system (AgilentTechnologies, CA, USA). The transcriptomes were sequenced using an Illumina HiSeq 3000 sequencer at Novogene Corporation (Tianjin, China). HTSeq v0.6.1 was used to count the reads numbers mapped to each gene. And then FPKM of each gene was calculated based on the length of the gene and reads count mapped to this gene. FPKM, expected number of Fragments Per Kilobase of transcript sequence per Millions base pairs sequenced, considers the effect of sequencing depth and gene length for the reads count at the same time, and is currently the most used method for estimating gene expression levels. The resulting *P*-value is adjusted using the Benjamini and Hochberg methods to control the error discovery rate. The corrected *P*-value < 0.05 was set as the threshold of significant differential expression.

### 2.4 Molecular simulation analyses

Protein sequences were aligned using CLUSTALW2 (http://www.expasy.ch). Sequences were retrieved using the protein search algorithm at the National Center for Biotechnology Information (NCBI), and conserved NadD domains were identified via NCBI's CD-Search. Multiple sequence comparison was carried out using Clustal Omega (Madeira et al., 2019) and ESPript software (Xavier and Patrice, 2014). A homology model of NadD was constructed using Robetta (https://robetta.bakerlab.org/) and Discovery Studio 2.0 (BIOVIA., 2017). Molecular docking analyses of nicotinic acid mononucleotide (NaMN) and NA were performed using the CDOCKER protocol of Discovery Studio 2.0 (BIOVIA., 2017).

### 2.5 Computer-assisted directed evolution of NadD

Virtual saturation mutagenesis of NadD was performed using MaxFlow (https://maxflow.ilabpower.com). To identify variants with increased affinity for nicotinamide mononucleotide (NMN) and reduced affinity for NaMN, 17 residues Met15, Gly16, Gly17, Thr18, Phe19, His26, Thr113, Gly114, Ser168, Asp20, Pro21, Ile112, Tyr92, Thr93, Ile94, Trp124, and His125 were individually mutated, and their interactions with NMN were evaluated. Virtual variants with predicted improvement of NMN binding ability (ΔG total (kcal/mol) < 0) were selected as candidates for the next evaluation. Next, the mutational *nadD* gene was cloned into the pET15-b vector, which carries an N-terminal His-tag. Site-directed metagenesis was performed by incubating template DNA, dNTPs, and the appropriate mutagenic primers with Pfu DNA polymerase, in accordance with the manufacturer's instruction manual. Mutant plasmids were verified by DNA sequencing and transformed into *Escherichia coli* BL21 (DE3) for recombinant protein expression and purification. Intracellular concentrations of NAD^+^ were determined as previously described (Zhang et al., 2009).

### 2.6 Expression and purification of wild-type and variant NadDsg

The *nadDsg* gene, a *nadD* homolog, was amplified from *S. gilvosporeus* F607 genomic DNA using the primer pair *nadDsg* His-F/R (Supplementary Table 1) and then cloned into pMD18T to generate pMD18T-nadDsg. After confirmation by DNA sequencing, *nadDsg* from pMD18T-nadDsg was cloned into the expression vector pET-15b to construct pET-nadDsg, which was transformed into *E. coli* BL21 (DE3) to obtain *E. coli* BL21/pET-nadDsg. BL21/pET-nadDsg was cultured to an OD_600nm_ of 0.6–0.8, and NadDsg expression was induced by the addition of 0.1 mM isopropyl-D-1-thiogalactopyranoside. His_6_-NadDsg fusion proteins were purified by Ni-NTA chromatography (Sangon Biotech, Shanghai, China) (Fu et al., 2019). The *nadDsg* mutants were cloned and synthesized by Sangon Biotech, and the variant proteins expressed and purified using the same protocol.

### 2.7 Detection of enzymatic reactions *in vitro*

For enzymatic quantification, levels of NAMN, NMN, ATP, nicotinic acid adenine dinucleotide (NAAD), and NAD were measured using HPLC, as previously reported (Mehl et al., 2000). Briefly, the substrates and enzyme in reaction buffer (100 mM Tris-HCl, 2.0 mM MgCl_2_, pH 8.0) were incubated with 2 mM ATP at 37° C for the appropriate time interval, and 20-mL aliquots were analyzed by high-pressure liquid chromatography (HPLC). HPLC was performed on Agilent Series 1,200 system (Agilent Technologies, CA, USA) with a diode array ultraviolet detector set at 254 nm and a Zorbax Eclipse XDB-C18 column. Buffer A contained 100 mM KH_2_PO_4_ (aqueous), pH 7.5. Buffer B contained 80% 100 mM KH_2_PO4 (aqueous), pH 7.5, and 20% methanol. Elution conditions were as follows: 100% buffer A for 0 to 7 min; 100% buffer A to 100% buffer B in 7 to 8 min; and 100% buffer B for 8 to 13 min. The flow rate was 1 mL/min.

### 2.8 Construction of *nadD* mutant strains and effect on natamycin production

An additional *nadD* mutant strain was constructed via *att* integration, as previously described (Gongli et al., 2022). Briefly, *nadD* mutant genes were cloned downstream of the *ermE* promoter region in the integrative vector pSET152 (Sangon Biotech, Shanghai, China) to generate pSET152-nadD_M_, which was then introduced into the genome of *S. gilvosporeus* F607 via intergeneric conjugation using *E. coli* ET12567 as the donor strain. Nalidixic acid (500 μg) and apramycin (1 mg) were added, and the culture was incubated for a further 5 days to select the exconjugants. Successful conjugants were confirmed by colony PCR using primers nadD V-F1 and nadD V-R1, and by DNA sequencing. Wild type *nadDsg* was used as a control. Natamycin was quantified by HPLC analysis, as previously described (Wang et al., 2016).

## 3 Results

### 3.1 Organic nitrogen availability influences nicotinate/nicotinamide metabolism and natamycin production

**Figure 1:**
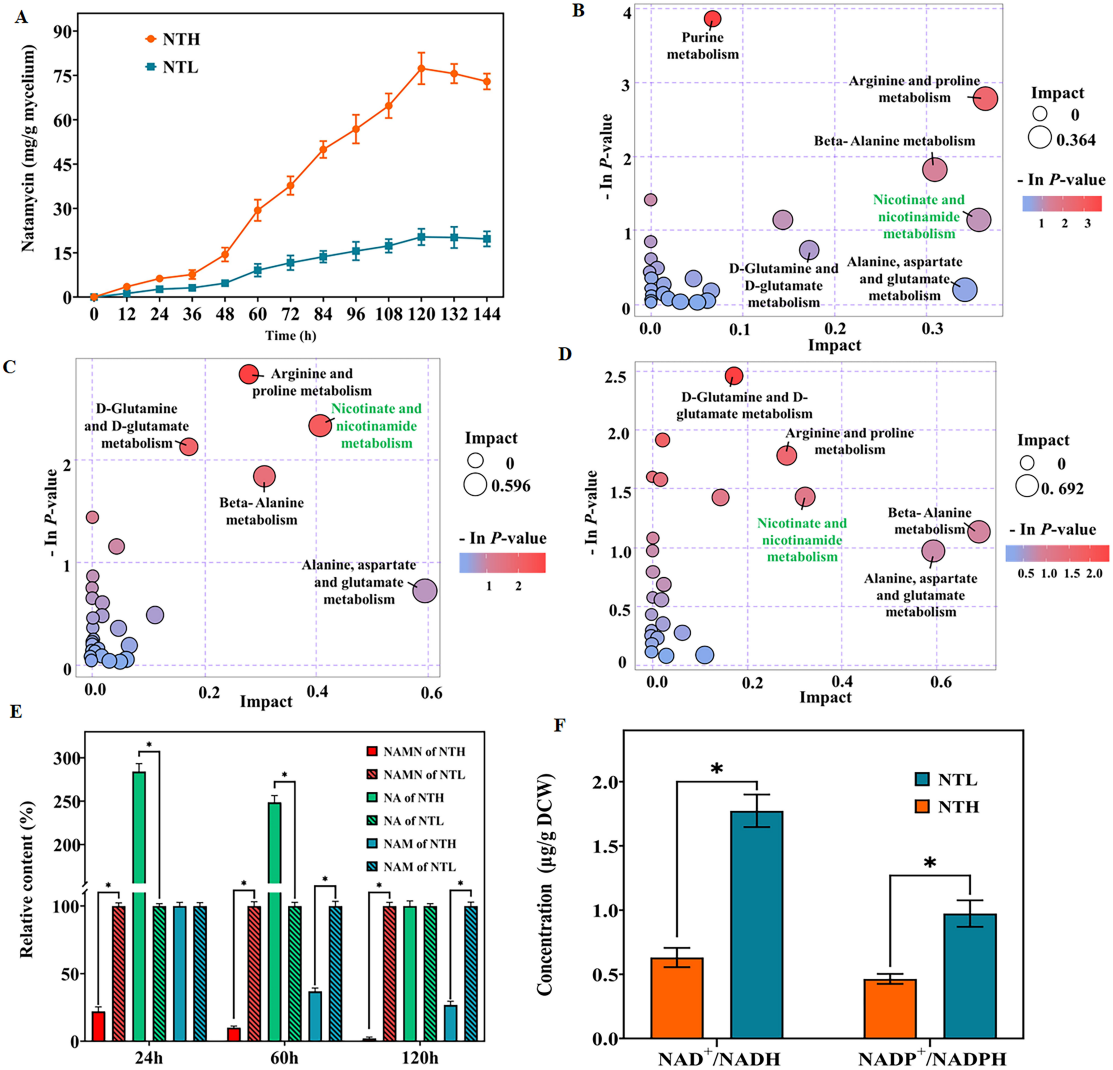
Influence of organic nitrogen availability on natamycin production and nicotinate/nicotinamide metabolism. **(A)** Natamycin production by *S. gilvosporeus* cultivated in high-nitrogen (NTH) and low-nitrogen (NTL) media over time. **(B–D)** Bubble diagrams of pathway analysis of the NTH and NTL groups at the early (24 h; B), rapid (60 h; C), and later (120 h; D) stages of natamycin biosynthesis. **(E)** Comparison of the levels of key metabolites of the nicotinate/nicotinamide metabolic pathway between the NTH and NTL groups at the three stages. The quantity of each metabolite in NTL group was defined as 100%. **(F)** Concentrations of total NAD^+^/NADH and NADP^+^/NADPH in the two groups at the rapid stage. ^*^ *p* < 0.05.

Nitrogen metabolism plays crucial roles in promoting natamycin production (Wang et al., 2025a). To investigate the impact of organic nitrogen content on natamycin biosynthesis, we cultured *S. gilvosporeus* F607 in two media formulations: NTH fermentation medium (20 g/L soy peptone and 4.5 g/L yeast extract) and NTL medium (2.0 g/L soy peptone and 0.45 g/L yeast extract). In NTL medium, containing low organic nitrogen, the maximum production of natamycin was 20.35 mg/g mycelium. In contrast, natamycin increased by 2.8-fold (reaching to 77.38 mg/g mycelium) when F607 was cultivated in NTH medium resulted (Figure 1A).

Metabolome analysis comparing intracellular profiles of F607 cultured in NTH and NTL media across the early (24 h), rapid (60 h), and later (120 h) stages of natamycin biosynthesis, revealed differential activity in nitrogen metabolism and several amino acid metabolism pathways, including arginine/proline, D-Glutamine/glutamate, beta-alanine, D-alanine histidine, and alanine/aspartate. Notably, metabolism of nicotinate and nicotinamide was identified across all three stages of natamycin biosynthesis and ranked among the top 10 pathways (Figures 1B–D).

Next, changes in three important metabolites in the nicotinate and nicotinamide metabolic pathway—NAMN, NAM (Niacinamide), and NA—were analyzed. At the early stage of natamycin biosynthesis, NAMN levels decreased (22.0%, *p* < 0.01), while NA was enriched (284.1%, *p* < 0.01) in the NTH cultivation group compared with that in the NTL group (Figure 1E). At the later stage, levels of both NAM and NAMN decreased (26.82% and 2.16%, respectively, *p* < 0.01) in the NTH group (Figure 1E). At the rapid stage, levels of NAMN and NAM were significantly lower (10.03% and 36.88%, *p* < 0.01) in the NTH group than in the NTL group (Figure 1E), indicating higher consumption of these precursors during high-yield natamycin biosynthesis.

NAM, NA, and NAMN are key intermediates in the biosynthesis of NADH and NADPH. Considering the demand for reducing power during natamycin biosynthesis, we quantified total NAD^+^/NADH and NADP^+^/NADPH concentrations at the rapid stage. Total NAD^+^/NADH (1.77 μg/g dry cell weight [DCW]) and total NADP^+^/NADPH (0.94 μg/g DCW) concentrations in the NTH cultivation group were higher than those in the NTL group (0.63 μg/g DCW and 0.46 μg/g DCW, respectively), representing 1.81-fold and 1.04-fold increases (Figure 1F). These findings indicated a directed metabolic flow from NAM and NAMN to NAD (H) and NADP(H) biosynthesis during rapid natamycin production under conditions of abundant organic nitrogen.

### 3.2 Abundant organic nitrogen affects genetic expression of the NAD^+^/NADP^+^ pathway during rapid natamycin biosynthesis

**Figure 2:**
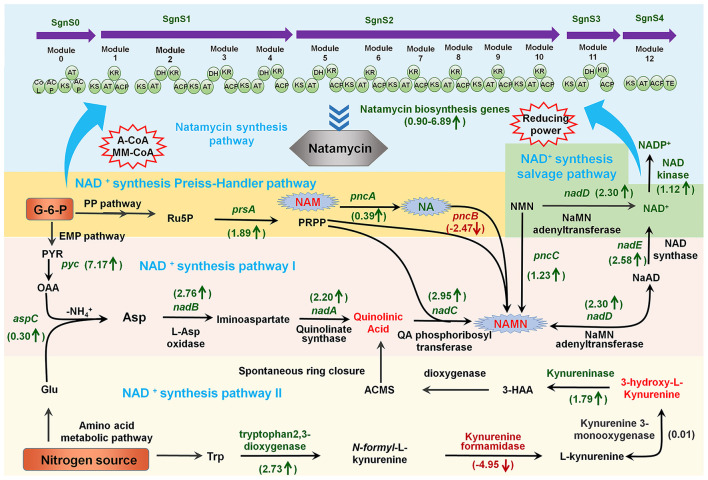
Schematic overview of the links between metabolites and the expression profiles of genes involved in NAD^+^/NADP^+^ biosynthesis and natamycin production in *S. gilvosporeus* F607. Metabolite and gene expression levels represent the results of metabolomic and transcriptomic data from the rapid phase (60 h) of natamycin biosynthesis. Metabolites and genes shown in red were downregulated in high-nitrogen medium compared with that in low-nitrogen medium (NTH/NTL), those in green were upregulated (NTH/NTL), and those in black showed no difference. Numerical values represent log_2_-fold changes of NTH60/NTL60.

To investigate the mechanism by which organic nitrogen stimulates NAD^+^/NADP^+^ and natamycin production in *S. gilvosporeus* F607, we conducted a transcriptome analysis. This revealed upregulation of all genes in the natamycin biosynthetic cluster. Notably, the PKS genes *sgnS4, sgnS3, sgnS2*, and *sgnS0* exhibited 5.1- to 6.1-fold increases in expression (log_2_ fold-change) in the NTH cultivation group compared with that in NTL group during the rapid stage of natamycin biosynthesis (Figure 2 and Supplementary Table 2).

In addition to two *de novo* NAD^+^ biosynthesis pathways (Ding et al., 2021) and a salvage pathway (Wang et al., 2017), a homolog of the Preiss–Handler pathway (Turło et al., 2012) has also been identified in *S. gilvosporeus* F607 (Figure 2), based on the genomic sequence (Genebank NO. CP020569). Within the Asp-derived synthesis pathway I illustrated in Figure 2, the NTH group showed upregulation of all eight key genes, including those encoding Asp oxidase (*nadB*), quinolinate synthase (*nadA*), QA phosphoribosyl transferase (*nadC*), NMN/NaMN adenylyltransferase (*nadD*), and NAD synthase (*nadE*), compared with the NTL group (Supplementary Table 3), leading to extensive consumption of QA and NAMN. In the Trp-derived pathway II (Figure 2) comprising five key genes, the expression levels of genes encoding tryptophan 2,3-dioxygenase and kynureninase was upregulated while those encoding kynurenine formamidase and kynurenine 3-monooxygenase were downregulated (Supplementary Table 3). Compared with the high expression across all genes in pathway I, the downregulation of two enzymes in pathway II may limit its NAD^+^ contribution. Therefore, *S. gilvosporeus* F607 appears to favor pathway I for NAD^+^ production during the rapid stage of natamycin biosynthesis.

In the Preiss–Handler pathway (Figure 2), expression of the key entry gene *pncB*, which facilitates the incorporation of NA into NAD^+^ biosynthesis, was significantly downregulated, whereas *pncA* was upregulated (Supplementary Table 3). The high expression of *pncA* leads to NAM reduction, while the low expression of *pncB* results in NA accumulation. In the salvage pathway involving NAD^+^ synthesis from NMN (Figure 2), *nadD* was upregulated (Supplementary Table 3). NAD can be synthesized via the amidated pathway, where NMN adenylyltransferase (NMNAT; a NadD homolog) is the key enzyme that directly converts NMN to NAD at the expense of ATP. This route represents an efficient mechanism for NAD^+^ production. However, strain F607 does not carry a gene encoding the nicotinamide phosphoribosyl transferase (NAMPT) enzyme, preventing the direct synthesis of NMN.

In summary, the metabolomic and transcriptomic analyses revealed a link between abundant organic nitrogen, preferential utilization of pathway I for NAD^+^/NADP^+^ synthesis, and rapid natamycin biosynthesis. Increasing NAD^+^ availability through NMN supplementation or modification of the salvage pathway represents a promising strategy for enhancing natamycin biosynthesis.

### 3.3 NMN supplementation promotes natamycin biosynthesis

**Figure 3:**
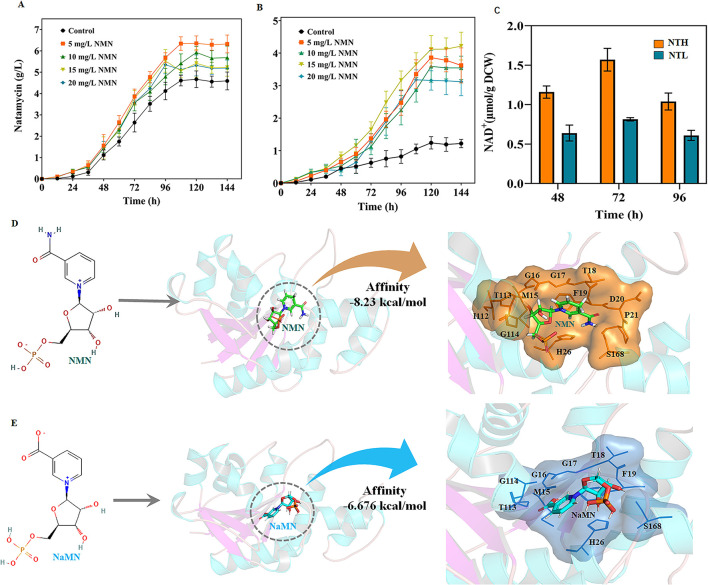
NMN supplementation promotes natamycin biosynthesis and NadDsg interactions with different substrates. **(A, B)** Natamycin production by *S. gilvosporeus* F607 cultivated under different levels of NMN supplementation in NTH **(A)** and NTL **(B)** media. **(C)** Concentrations of intracellular NAD^+^ in F607 cells cultivated in in NTH or NTL media. **(D, E)** Molecular simulations of NadDsg interactions with NMN **(D)** and NaMN **(E)**. Substrate-binding sites are represented by color shadowing. Binding amino acid residues are represented by orange (NMN) and blue (NaMN) lines.

Because *S. gilvosporeus* F607 does not carry genes encoding NMN synthesis enzymes (e.g., NAMPT and NMN synthase), it cannot synthesize NMN like most microbes (Xianzhong et al., 2014). However, NMN supplementation of the culture medium is a strategy for enhancing NAD^+^ and natamycin production in the presence of *nadD* in this strain. To further evaluate the impact of NMN on natamycin fermentation, it was added to NTL and NTH media at intervals of 5 mg/L final concentration (0–20 mg/L). At 120 h, the natamycin concentration in F607 was ~35.97% higher in NTH medium supplemented with 5.0 mg/L NMN than in non-supplemented medium (Figure 3A). Under low organic carbon source conditions, the natamycin concentration was ~2.31-fold higher in F607 cells grown for 120 h in NTL medium containing 15 mg/L NMN than in cells grown in NTL medium alone (Figure 3B). Compared with NTL medium, the cells grown in NTH showed greater regeneration of intracellular NAD^+^ (Figure 3C), with a 1.87-fold (1.57 μmol/g DCW) increase at 72 h. These results confirmed that NMN supplementation could increase NAD^+^ concentrations, thereby improving natamycin biosynthesis.

The NMN/NaMN adenylyltransferase encoded by *nadD*, collectively referred to as pyridine nucleotide adenylyltransferase, is an indispensable enzyme that catalyzes the central step of all NAD biosynthesis pathways, recognizing both NMN and NaMN as substrates (Zhang et al., 2003). Considering that the structural and catalytic properties of NadD have not been characterized in *S. gilvosporeus*, we examined the molecular features and substrate specificity of the novel enzyme NadDsg in strain F607. NadDsg was found to contain a central six-stranded parallel β-sheet flanked by eight helices in a typical Rossmann fold topology (Zhang et al., 2003) (Supplementary Figure 1). In *E. coli*, NadD strongly favors NaMN over NMN, catalyzing the formation of NAAD with 20-fold greater efficiency than NAD (Mehl et al., 2000; Wang et al., 2017). NadDsg showed only a slight preference for NMN (-8.23 kcal/mol affinity) over NaMN (-6.676 kcal/mol affinity) (Figures 3D, E).

In molecular simulation analysis, NadDsg was revealed to bind to NaMN through nine amino acid residues: Met15, Gly16, Gly17, Thr18, Phe19, His26, Thr113, Gly114, and Ser168 (Figure 3D). These residues are consistent with the conserved GxFxPx[H/T]xxH and ISSTxxR motifs reported across humans, yeast, nematodes, and bacteria (Lau et al., 2009). Additionally, Asp20, Pro21, and Ile112 were predicted to contribute to substrate binding during NadDsg–NMN interaction (Figure 3E), while His26 forms stacking interactions with the pyridine ring and plays a critical role in NaMN recognition (Supplementary Figure 2). These findings suggested that, among the binding residues, Asp20, Pro21, and Ile112 may play major roles in substrate recognition by NadDsg.

### 3.4 NadDsg mutant constructions and screening with higher NMN preference

**Figure 4:**
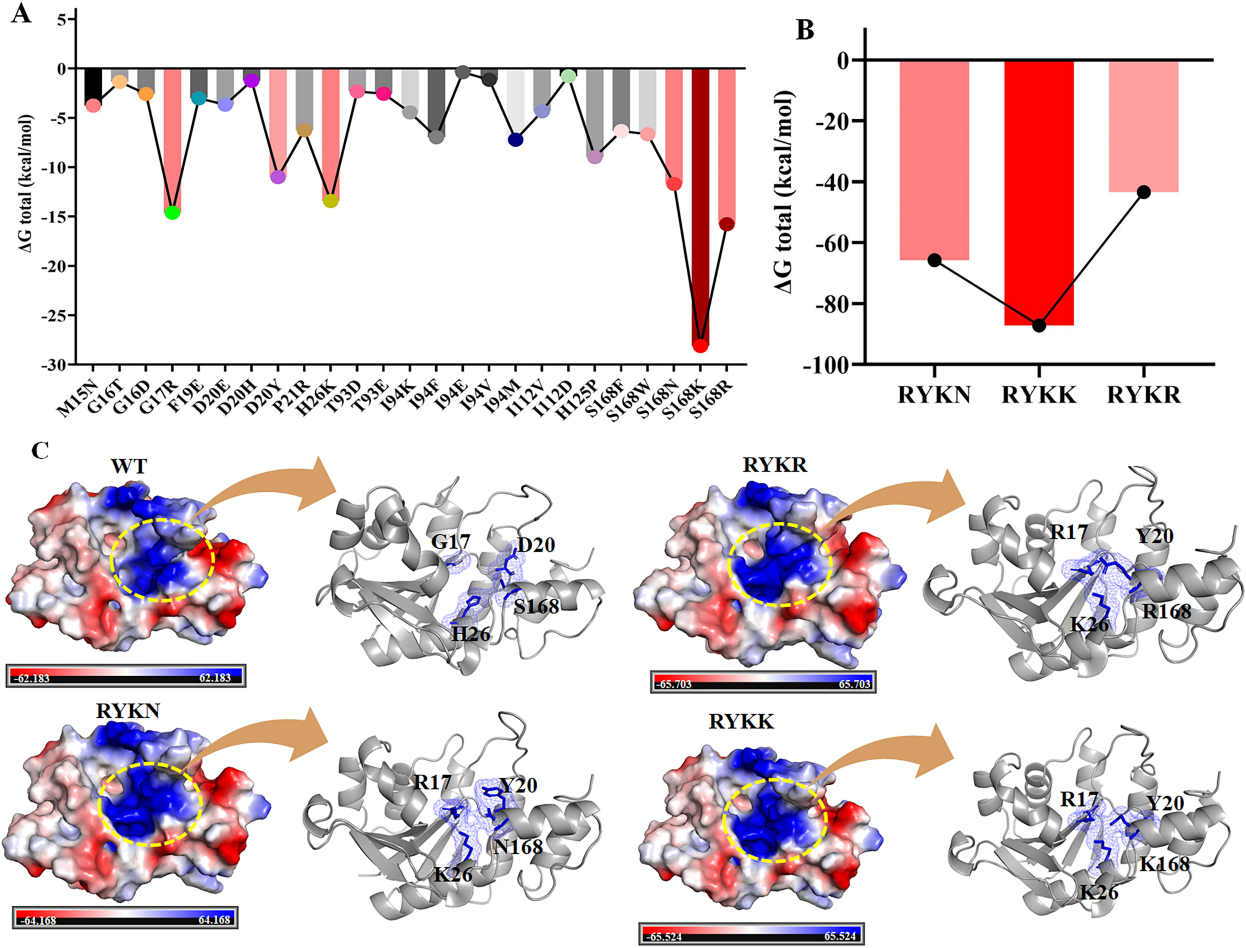
NadDsg mutations aimed at strengthening its preference for NMN. **(A, B)** ΔG energy variations of single-point **(A)** and combination mutations **(B)** of NadDsg. **(C)** Electrostatics of wild-type (WT) NadDsg and its RYKK/N/R mutants. Amino acid residues are represented by blue sticks.

The above results indicate that NadDsg with higher NMN preference would be a convenient and fast way to enhance NAD^+^ and natamycin biosynthesis in F607 in the presence of NMN addition. To alter the substrate preference, we chose to make changes at 17 residues: Met15, Gly16, Gly17, Thr18, Phe19, His26, Thr113, Gly114, Ser168, Asp20, Pro21, Ile112, Tyr92, Thr93, Ile94, Trp124, and His125. Single-point virtual saturation mutagenesis of these amino acids yielded 338 virtual variants, among which 25 exhibited varying degrees of improved NMN binding ability (Figure 4A and Supplementary Table 4). Among the variants with significantly impacted binding ability, more than half (14) were located within the conserved GxFxPx[H/T]xxH and ISSTxxR motifs. Notably, the S168K mutant exhibited the most pronounce effect, with a total ΔG energy variation of −28.12 kcal/mol (Figure 4A). Additionally, the D20Y, S168N, H26K, G17R, and S168R mutants presented total ΔG energy variations of −11 kcal/mol, −11.69 kcal/mol, −13.43 kcal/mol, −14.61 kcal/mol, and −15.78 kcal/mol (Figure 4A), respectively, which were attributed to variations in van der Waals forces (ΔE_vdw_) and electrostatic interaction (ΔE_ele_) (Supplementary Table 4). These findings indicated a significant role of S168 in NadDsg–NMN binding.

Next we evaluated the combined effects of G17**R**, D20**Y**, and H26**K** with three S168 point mutations (S168**R**, S168**K**, and S168**N**), designated R^17^Y^20^K^26^R^168^ (RYKR), R^17^Y^20^K^26^K^168^ (RYKK), and R^17^Y^20^K^26^N^168^ (RYKN), respectively. Among the S168 single variants, enzyme binding strength was ranked as S168K > S168R > S168N (Figure 4A). The total ΔG of the combined mutations RYKK and RYKN exceeded the sum of the corresponding single-point mutations, indicating synergistic effects. In contrast, the ΔG change of RYKR was less than the cumulative impact of its individual point mutations (Figure 4B and Supplementary Table 5). Overall, the binding strength of these combination mutants followed the order RYKK > RYKN > RYKR.

Kinetic analysis indicated that, compared with wild-type NadDsg, all three combination mutants had lower *K*_*m*_ values and higher *k*_*cat*_ values for NMN (Table 1). The catalytic efficiency (*k*_*cat*_/*K*_*m*_) of the mutants with respect to NMN was increased by 52.9-fold, 5.36-fold, and 9.36-fold, respectively, relative to NadDsg (Table 1). Additionally, the NadDsg_RYKK_ mutant exhibited an 55.26-fold increase in selectivity for NMN over NaMN (from 1.49 to 82.30) (Table 2), indicating a shift in substrate specificity and enhanced preference for NMN.

**Table 1:** Differential expression of natamycin biosynthesis PKS genes and NAD^+^/NADP^+^ biosynthesis genes.

**Natamycin biosynthesis PKS genes**			**NAD**^**+**^**/NADP**^**+**^ **biosynthesis genes**		
**Gene id**	**Gene Description**	**log2FoldChange (NTH/NTL)**	**id**	**Gene Description**	**log2FoldChange (NTH/NTL)**
B1H19_RS05020	SgnS4	5.698460908	B1H19_RS10955	NAD kinase	1.121823625
B1H19_RS05025	SgnS3	5.126958996	B1H19_RS12720	NadA	2.201256121
B1H19_RS05030	SgnS2	5.591874351	B1H19_RS14905	NadD	2.300976653
B1H19_RS05075	SgnS0	6.106795442	B1H19_RS27920	NadE	2.577468633
B1H19_RS05085	SgnS1	4.424676334	B1H19_RS29395	PncC	1.229274518

**Table 2:** Kinetic parameters of wild-type and combination mutants of NadDsg.

**Mutants of NadDsg**	**Kinetics for NMN**			**Kinetics for NaMN**			**Selectivity (*k_*cat*_*/*K_*m*_* with NMN)/(*k_*cat*_*/*K_*m*_* with NaMN)**
	***K**_*m*_* **(mM)**	***k**_*cat*_* **(s** ^−1^ **)**	***k**_*cat*_***/*****K**_*m*_***(mM**^−1^ **s**^−1^**)**	***K**_*m*_* **(mM)**	***k**_*cat*_* **(s** ^−1^ **)**	***k**_*cat*_***/*****K**_*m*_***(mM**^−1^ **s**^−1^**)**	
NadDsg	1.49	2.61	1.75	2.18	2.57	1.18	1.49
NadDsg_RYKK_	0.11	12.12	110.18	3.72	4.98	1.34	82.30
NadDsg_RYKN_	0.47	5.24	11.15	9.17	6.69	0.73	15.22
NadDsg_RYKR_	0.34	6.17	18.15	5.31	16.26	3.06	5.93

Evaluation of the substrate-binding preferences of the combination mutants revealed that the RYKK/N/R mutations altered the binding pocket by inducing bending and extension (Figure 4C), leading to more stringent substrate selection and reduced accessibility. Compared to the NadDsg (wild type), NadDsg_RYKK_, NadDsg_RYKN_, and NadDsg_RYKR_ changed the conformation of NMN binding cavity that composed by four amino acid residues (Supplementary Figure 3). In NadDsg, G17, D20, H26 and S168 composed a nearly cubic spatial structure (6.4Å × 6.2Å × 5.4Å × 4.8Å). In mutants, all distances were shortened and led to a distorted structure, especially in NadDsg_RYKK_, elongated spatial structure (4.0Å × 6.0Å × 3.6Å × 3.2Å) may cause an alternative bent and extended NMN conformations, which was reported to be a catalytic mechanism of NMN adenylyltransferase in Human (Zhang et al., 2003). Moreover, compared with wild-type NadDsg, the mutations in NadDsg_RYKK_, NadDsg_RYKN_, and NadDsg_RYKR_ all enhanced the electrostatics to different degrees (Figure 4C). Pyridine nucleotide adenylyltransferase has been proposed to use an in-line nucleophilic attack of the NMN phosphate on the ATP α-phosphate (Zhang et al., 2003). These enhanced electrostatics provided a more suitable chemical environment for nucleophilic attack and a stronger preference for NMN.

### 3.5 Supplementation of NMN and NadDsg_RYKK_ overexpression promotes natamycin biosynthesis

**Figure 5:**
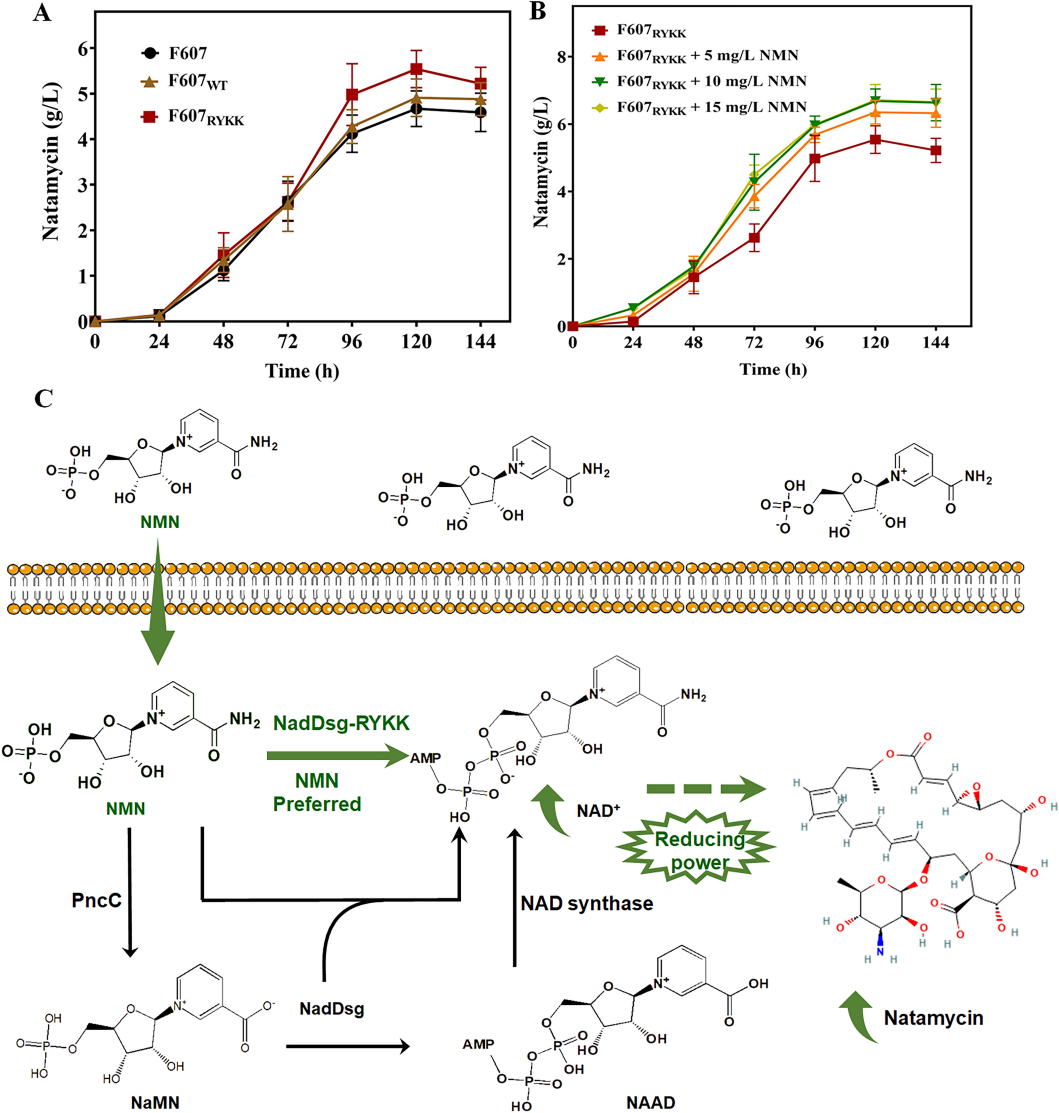
Enhancement of natamycin biosynthesis through NMN supplementation combined with NadDsg_RYKK_ overexpression. **(A, B)** Natamycin production over time in three *S. gilvosporeus* F607 strains cultivated in NTH medium containing non **(A)** and 0-15 mg/L **(B)** NMN in original F607 (F607), F607 overexpressing wild-type NadDsg (F607_WT_), and F607 overexpressing NadDsg_RYKK_ (F607_RYKK_). **(C)** Schematic representation of the strategy used to enhance natamycin biosynthesis. Green represent the modified NMN preferred pathway of this study, black represent the original metabolic pathway.

The integrated transcriptomic and metabolomic analyses suggested that NMN supplementation had a positive influence on natamycin biosynthesis in F607. To further increase natamycin production, a strain with overexpression of the NadDsg_RYKK_ mutant (F607_RYKK_) was constructed and cultivated in NTH medium supplemented with no NMN supplementation. At 120 h, natamycin production in strain F607_RYKK_ reached 5.24 g/L, compared with 4.67 g/L in strain F607, representing a 1.12-fold (*p* < 0.05) enhancement (Figure 5A).

Then, NMN was added to NTH medium at a final concentration of 5–15 mg/L. At 120 h, natamycin production by F607_RYKK_ reached 6.68 g/L in NTH medium supplemented with 10 mg/L NMN (increase by 0.275-fold, *p* < 0.05), compared with no NMN supplementation (5.24 g/L) (Figure 5B). Relative to F607 (4.67 g/L), NadDsg_RYKK_ overexpression combined with 10 mg/L NMN supplementation led to a 43.1% increase (*p* < 0.05) increase in natamycin production (Figure 5B). Additionally, natamycin initiation occurred earlier (12–24 h) in the NadDsg_RYKK_ overexpression strain, leading to increased demand for NMN. In the wild-type strain F607, 5 mg/L NMN represented the optimal supplementation level for natamycin biosynthesis (Figure 3A). In contrast, F607_RYKK_ required 10 mg/L NMN to reach its maximum natamycin production level, potentially reflecting differences in processing capacity between the native NadDsg and engineered NadDsg_RYKK_. These results indicated that NMN supplementation combined with NadDsg_RYKK_ overexpression is an effective strategy to enhance natamycin biosynthesis (Figure 5C). Gaining a deeper understanding of the functional roles of these motifs will inform future engineering strategies aimed at enhancing natamycin production.

## 4 Discussion

Natamycin, a glycosylated 26-membered tetraene macrolide antifungal produced by multiple *Streptomyces* species, has become a major mold inhibitor in the food industry (Yangla and Yldz, 2016) and has shown potential for inhibiting cancer cell proliferation (Vasquez et al., 2020). Approaches to improve natamycin productivity have included optimization of large-scale fermentation, classical random mutagenesis, genetic engineering (Aparicio et al., 2016), and introduction of chemical or biological elicitors (Zong et al., 2021). In addition to adequate amounts and types of carbon, nitrogen metabolism was confirmed as a key factor in the production of natamycin (Farid et al., 2000). Recently, overexpression of the nitrogen metabolism regulatory gene *glnR* in *S. gilvosporeus* was shown to increase natamycin production, achieving a final yield 1.67 times higher than that of the original strain (Wang et al., 2025b). In this study, our findings suggest that an abundance of organic nitrogen promotes natamycin production by enhancing the nicotinate/nicotinamide metabolic pathway, which may serve as a source of reducing power necessary for natamycin biosynthesis. Additionally, rapid biosynthesis of natamycin created high demand for NAD(H) and NADP(H). Accordingly, strategies that bolster reducing power may provide a viable means to increase natamycin yield.

For *Streptomyces*, amino acid metabolism and NAD synthesis are primary metabolism, which provide the precursors of secondary metabolism (Rodríguez et al., 2018). *S. gilvosporeus* F607 preferred the aspartic acid pathway rather than the tryptophan-Kynurenic acid pathway to synthesize NAD(P) in rich nitrogen. That may relate to the crosstalk between carbon, nitrogen regulation of secondary metabolism. It was reported that factors (such as ArgR and GlnR) linked the arginine and carbon metabolism (Rodríguez et al., 2018). In this study, we proposed that, to provide sufficient precursors for natamycin metabolism, *S. gilvosporeus* linked the metabolism of carbon and nitrogen through the Asp pathway (glutamic acid and oxaloacetic acid condensation) under high nitrogen source conditions.

Previously, 0.0025% (w/v) nicotinamide and 0.005% (w/v) NA, defined as growth promoters, were shown to enhance tacrolimus production in *S. tsukubaensis* by ~3-fold and ~6-fold, respectively, probably by stimulating NAD/NADP biosynthesis (Turło et al., 2012). Furthermore, upregulation of glycolysis and the PPP pathway increases the supply of pyruvate and NADPH, which are essential for the synthesis of acetyl-CoA and malonyl-CoA, as well as for providing reducing power during natamycin biosynthesis (Wang et al., 2025b). Here, integrated transcriptomic and metabolomic analyses of *S. gilvosporeus* F607 also revealed a link between natamycin biosynthesis and nicotinate/nicotinamide metabolism, along with significant enhancement of the NAD biosynthesis pathway originating from Asp.

In *E. coli, de novo* NAD is synthesized via the deamidated or amidated pathways. In the deamidated pathways, NaMN is coupled with ATP by the action of NaMN adenylyltransferase to form NAAD, which is amidated to give NAD by the action of NAD synthetase encoded by *nadE* (D'Angelo et al., 2000). Alternatively, in the amidated pathway, NAD can be synthesized via NMN adenylyltransferase catalysis, which directly converts NMN to NAD at the expense of ATP (Magni et al., 2009). However, the NMN adenylyltransferase in *E. coli*, NadD, strongly favors NaMN over NMN (Mehl et al., 2000). In *S. gilvosporeus* F607, we investigated the NAD biosynthetic pathway and the substrate preference of the NadD homolog NadDsg. NadDsg exhibited a slightly higher affinity for NMN (-8.23 kcal/mol) compared with NaMN (-6.676 kcal/mol), favoring a one-step catalytic path for NAD synthesis via the deamidated pathway. This preference may contribute to greater catalytic efficiency relative to the two-step amidated pathway. This metabolic paradox that *S. gilvosporeus* F607 does not carry a NAMPT enzyme to synthesize NMN but can use NMN by NadDsg to synthesize NAD may be related to two points. *S. gilvosporeus* is a soil microorganism, it need gain a competitive advantage by inhibit the growth of fungi or other bacteria (Evan et al., 2023). On the one hand, losing the ability to synthesize NMN during their evolution, may making their metabolism more efficient and rapid. On the other hand, transporting NMN from the soil environment is an economical and practical way for *S. gilvosporeus* form other NMN producing species, like plants, animals, or microorganisms (Shen et al., 2021). More investigations on the NMN transporter (encoded by B1H19_RS09230 gene in F607 genome) may be another effective strategy for enhancing the synthesis of natamycin.

Furthermore, the engineered mutant NadDsg_RYKK_ demonstrated an enhanced affinity for NMN. Given that most microbial cells lack the pathway required to synthesize NMN, NMN supplementation was applied to the NadDsg_RYKK_ overexpression strain F607_RYKK_ to boost natamycin production. At 120 h, the natamycin yield of F607_RYKK_ increase by a 43.1% increase, reached 6.68 g/L, compared with wild-type F607, confirming the efficacy of this strategy to enhance natamycin biosynthesis. Additionally, further researches on analysis of kinetic parameters of mutant enzymes and applicability of the production platform to other strains are needed.

## 5 Conclusion

This study elucidates a previously unrecognized regulatory mechanism linking organic nitrogen availability to natamycin biosynthesis in *S. gilvosporeus* F607 through NAD (P) cofactor elevation. Cultivation in organic nitrogen-rich medium enhanced NAD (P) pathway metabolites and established NAD(P) cofactor abundance as a critical mediator of nitrogen-driven secondary metabolism. The discovery and functional characterization of the NadDsg, which preferentially utilizes NMN over NaMN, provided a mechanistic target for metabolic engineering. Strategic supplementation with 10 mg/L NMN combined with overexpression of the engineered NadDsg_RYKK_ variant (with enhanced substrate specificity) synergistically boosted natamycin production to 6.68 g/L – a 43.1% improvement over the wild-type strain. The yield improvement through combined metabolic and enzyme engineering validates the translational potential of these findings for industrial-scale natamycin production.

## Data Availability

The original contributions presented in the study are included in the article/Supplementary material, further inquiries can be directed to the corresponding author.
