# Supplementary material for: Abundant organic nitrogen enhances natamycin biosynthesis by increasing NAD(P) metabolic pathway activity in Streptomyces gilvosporeus F607
Source: Front Microbiol. 2025 Oct 27;16:1684019. doi: 10.3389/fmicb.2025.1684019 (PMC12599879; doi:10.3389/fmicb.2025.1684019)
Supplement: Supplementary file 1 [file Table_1.docx]

Supplementary Material

# Supplementary Figures and Tables

## Supplementary Figures

**
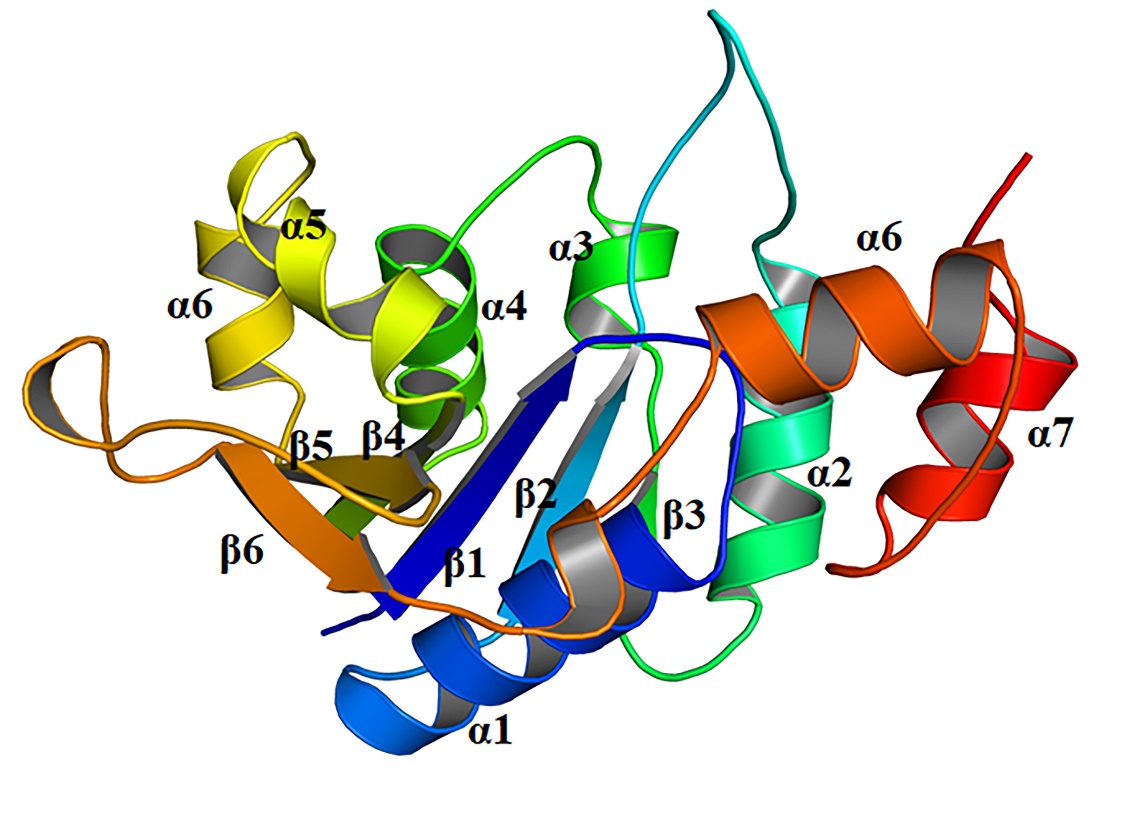
**

**Supplementary Figure S1.** Overall structure of the NadDsg from *S. gilvosporeus* F607.


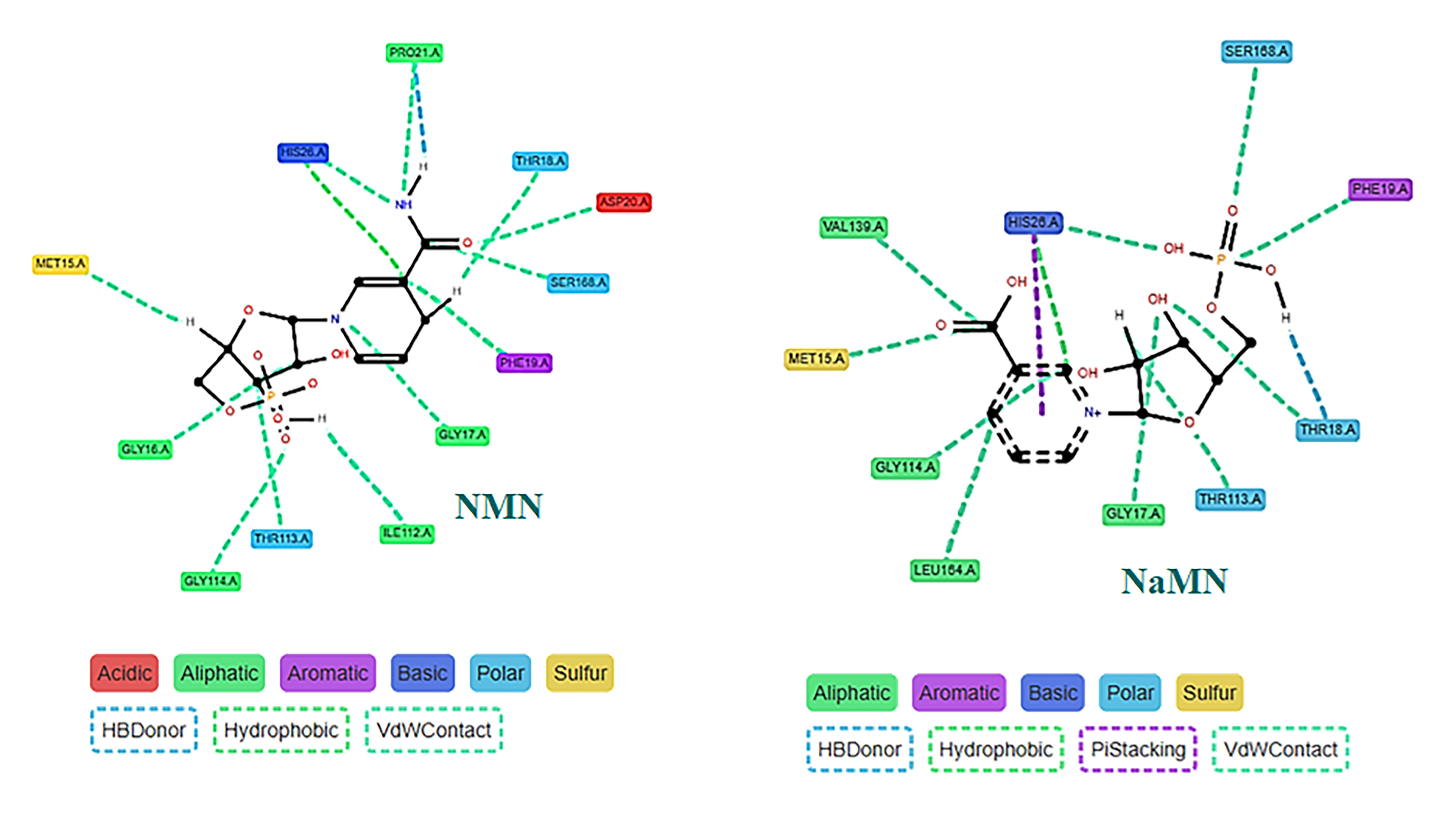


**Supplementary Figure S2.** Predicted NMN and NaMN binding amino acids in NadDsg. NMN and NaMN are shown as black sticks; binding sites are shown by lines.


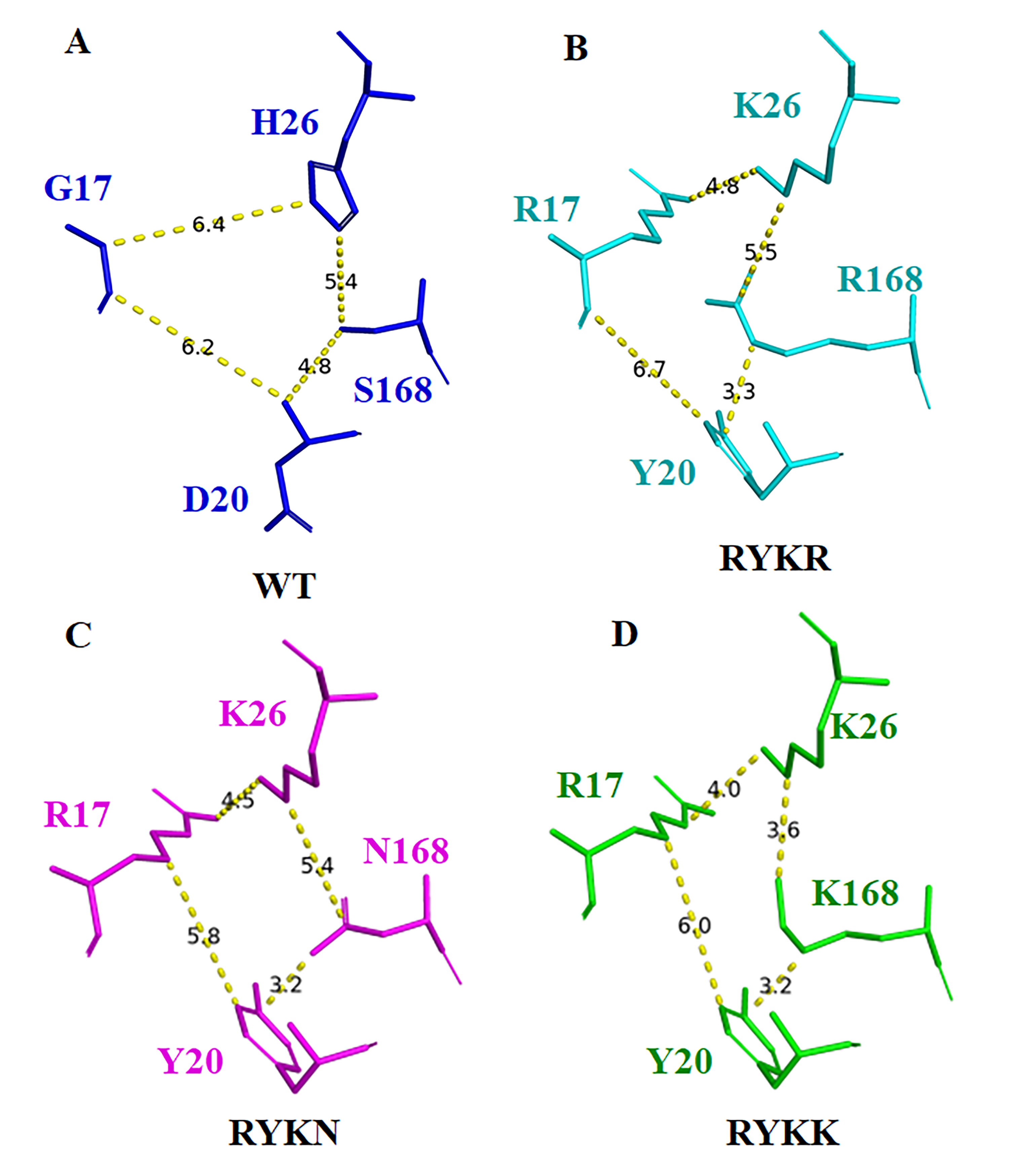


**Supplementary Figure S3.** The relative positions of the G17, D20, H26 and S168 that contribute to the formation of the substrate binding pocket. Amino acid residues are shown as colored sticks; binding sites are shown by lines. The dashed lines and numbers indicate relative distances (Å).

## Supplementary Tables

**Supplementary TableS1** Strains, plasmids and primers used in this study

| **Strains and plasmids** | | Description | |
| --- | --- | --- | --- |
| *S. gilvosporeus* F607 | | A natamycin producing strain | |
| *S. gilvosporeus* F607_RYKK_ | | NadDsg-RYKK mutant overexpression strain based on *S. gilvosporeus* F607 | |
| *E. coli* DH5α | | Strain used for gene clone | |
| *E. coli* BL21(DE3) | | Strain used for protein expression | |
| *E. coli* ET12567 (pUZ8002) | | Non-methylating donor for conjugation between *E.coli* and *Streptomyces* | |
| **Plasmids** | |  | |
| pMD18-T | | General cloning vector | |
| pET-15b | | Plasmid for *sgnE* and *oxyR* gene expression in *E. coli* | |
| pSET152 | | *Streptomyces* integrative vector with Apramycin resistance | |
|  | |  | |
| **Primers** | | | Sequences (5’ →3’) |
| **For *nadDsg* expression** | | | |
| *nadDsg*-His-F | *catatg*ATGGGAGAGCACACAGGGCC (*NdeI*) | | |
| *nadDsg*-His-R | *ctcgag*TACCGCAGCGACCGCTGA (*XhoI*) | | |

**Supplementary TableS2** Differential expression of natamycin biosynthesis genes.

| **id** | **Gene Description** | **baseMean_TL60** | **baseMean_TH60** | **foldChange(TH60/TL60)(TH60/TL60)** | **log2FoldChange** |
| --- | --- | --- | --- | --- | --- |
| gene-B1H19_RS05005 | SgnM | 29.82322828 | 2182.819906 | 73.19193901 | 6.193612861 |
| gene-B1H19_RS05010 | SgnR | 608.0756282 | 3231.203816 | 5.313818982 | 2.409749083 |
| gene-B1H19_RS05015 | SgnK | 401.1882111 | 14579.90715 | 36.3418135 | 5.183558508 |
| gene-B1H19_RS05020 | SgnS4 | 2923.452456 | 151811.1596 | 51.92872535 | 5.698460908 |
| gene-B1H19_RS05025 | SgnS3 | 1503.875029 | 52550.90416 | 34.9436643 | 5.126958996 |
| gene-B1H19_RS05030 | SgnS2 | 5028.077704 | 242506.784 | 48.23051636 | 5.591874351 |
| gene-B1H19_RS05035 | SgnI | 436.6201212 | 7108.10157 | 16.27983051 | 4.025013774 |
| gene-B1H19_RS05040 | SgnJ | 856.8420875 | 18123.51561 | 21.15152357 | 4.402689681 |
| gene-B1H19_RS05045 | SgnA | 336.540717 | 860.5957622 | 2.557181698 | 1.354554673 |
| gene-B1H19_RS05050 | SgnB | 266.1807272 | 549.6427286 | 2.064923085 | 1.046088045 |
| gene-B1H19_RS05055 | SgnE | 845.8290647 | 100463.7621 | 118.7754905 | 6.892093355 |
| gene-B1H19_RS05060 | SgnC | 742.9846764 | 64983.00434 | 87.46210576 | 6.450586178 |
| gene-B1H19_RS05065 | SgnG | 429.1655413 | 33633.01644 | 78.36839915 | 6.292200122 |
| gene-B1H19_RS05070 | SgnF | 10.02879712 | 209.7297319 | 20.91275049 | 4.386310916 |
| gene-B1H19_RS05075 | SgnS0 | 946.2466327 | 65212.81573 | 68.91735567 | 6.106795442 |
| gene-B1H19_RS05080 | SgnL | 80.44854835 | 400.1224331 | 4.973643917 | 2.314303223 |
| gene-B1H19_RS05085 | SgnS1 | 3230.133919 | 69371.45855 | 21.47634132 | 4.424676334 |
| gene-B1H19_RS05090 | SgnD | 309.0386169 | 26820.58143 | 86.78715205 | 6.439409577 |
| gene-B1H19_RS05120 | SgnT | 126.9422295 | 237.6886653 | 1.87241603 | 0.904901021 |

**Supplementary TableS3** Differential expression of NAD^+^ biosynthesis genes.

| **id** | **Gene Description** | **baseMean_TL60** | **baseMean_TH60** | **foldChange(TH60/TL60)(TH60/TL60)** | **log2FoldChange** |
| --- | --- | --- | --- | --- | --- |
| gene-B1H19_RS03570 | PncB | 216.5157282 | 39.200419 | 0.1810511 | -2.465530865 |
| gene-B1H19_RS10955 | NAD kinase | 106.9980535 | 232.85118 | 2.1762188 | 1.121823625 |
| gene-B1H19_RS12720 | NadA | 1547.956403 | 7118.7353 | 4.5987957 | 2.201256121 |
| gene-B1H19_RS14905 | NadD | 138.0810924 | 680.45155 | 4.9279125 | 2.300976653 |
| gene-B1H19_RS16185 | PncA | 83.80358953 | 109.66009 | 1.3085369 | 0.387954631 |
| gene-B1H19_RS17280 | pyc | 778.2464809 | 112044.99 | 143.97108 | 7.169635226 |
| gene-B1H19_RS18170 | Kynurenine formamidase | 186.9776893 | 6.0480825 | 0.0323465 | -4.950244498 |
| gene-B1H19_RS20195 | Kynureninase | 265.0458177 | 919.27606 | 3.4683666 | 1.794256398 |
| gene-B1H19_RS20200 | Tryptophan2,3-dioxygenase | 211.7056705 | 1410.344 | 6.6618148 | 2.735915247 |
| gene-B1H19_RS23590 | NadB | 217.888018 | 1477.562 | 6.7812908 | 2.761559905 |
| gene-B1H19_RS23595 | NadC | 192.9992789 | 1487.1947 | 7.7057009 | 2.945926188 |
| gene-B1H19_RS27920 | NadE | 233.3192777 | 1392.6629 | 5.9689147 | 2.577468633 |
| gene-B1H19_RS29395 | PncC | 63.96228839 | 149.95899 | 2.3444906 | 1.229274518 |
| gene-B1H19_RS29560 | kynurenine 3-monooxygenase | 141.1375832 | 142.28432 | 1.008125 | 0.011674473 |
| gene-B1H19_RS34915 | AspC | 201.7816265 | 249.04227 | 1.2342168 | 0.303595809 |
| gene-B1H19_RS04210 | prsA | 184.5494602 | 684.4343554 | 3.708677092 | 1.89090466 |

**Supplementary TableS4** Assessment of NadDsg single-point virtual saturation mutagenesis by MaxFlow.

| **structure_name** | **ΔG total (kcal/mol)** | **ΔE vdw (kcal/mol)** | **ΔE ele (kcal/mol)** | **ΔE polar (kcal/mol)** | **ΔE non-polar (kcal/mol)** | **ΔG gas (kcal/mol)** | **ΔG solv (kcal/mol)** |
| --- | --- | --- | --- | --- | --- | --- | --- |
| 125HIS_PRO | -8.98(0.00) | -32.13(0.00) | -9.42(0.00) | 37.33(0.00) | -4.76(0.00) | -41.55(0.00) | 32.57(0.00) |
| 94ILE_MET | -7.22(0.00) | -31.49(0.00) | -8.62(0.00) | 37.65(0.00) | -4.77(0.00) | -40.11(0.00) | 32.88(0.00) |
| 94ILE_PHE | -6.97(0.00) | -31.38(0.00) | -8.57(0.00) | 37.74(0.00) | -4.77(0.00) | -39.94(0.00) | 32.97(0.00) |
| 168SER_TRP | -6.65(0.00) | -32.84(0.00) | 6.14(0.00) | 24.80(0.00) | -4.76(0.00) | -26.69(0.00) | 20.04(0.00) |
| 168SER_PHE | -6.33(0.00) | -34.37(0.00) | 5.35(0.00) | 27.47(0.00) | -4.78(0.00) | -29.02(0.00) | 22.69(0.00) |
| 21PRO_ARG | -6.25(0.00) | -24.83(0.00) | -84.39(0.00) | 107.73(0.00) | -4.76(0.00) | -109.22(0.00) | 102.97(0.00) |
| 94ILE_LYS | -4.44(0.00) | -30.48(0.00) | -35.64(0.00) | 66.44(0.00) | -4.76(0.00) | -66.12(0.00) | 61.68(0.00) |
| 112ILE_VAL | -4.34(0.00) | -30.49(0.00) | -6.06(0.00) | 36.98(0.00) | -4.76(0.00) | -36.56(0.00) | 32.21(0.00) |
| 15MET_ASN | -3.76(0.00) | -29.34(0.00) | -4.68(0.00) | 34.95(0.00) | -4.69(0.00) | -34.02(0.00) | 30.26(0.00) |
| 20ASP_GLU | -3.67(0.00) | -30.38(0.00) | -4.38(0.00) | 35.85(0.00) | -4.76(0.00) | -34.76(0.00) | 31.09(0.00) |
| 19PHE_GLU | -3.01(0.00) | -30.17(0.00) | 68.67(0.00) | -36.61(0.00) | -4.90(0.00) | 38.50(0.00) | -41.51(0.00) |
| 168SER_LYS | -28.12(0.00) | -28.67(0.00) | -125.49(0.00) | 130.77(0.00) | -4.72(0.00) | -154.16(0.00) | 126.04(0.00) |
| 16GLY_ASP | -2.58(0.00) | -30.81(0.00) | 50.14(0.00) | -17.18(0.00) | -4.73(0.00) | 19.34(0.00) | -21.91(0.00) |
| 93THR_GLU | -2.56(0.00) | -30.02(0.00) | 39.50(0.00) | -7.28(0.00) | -4.76(0.00) | 9.48(0.00) | -12.04(0.00) |
| 93THR_ASP | -2.32(0.00) | -29.67(0.00) | 31.84(0.00) | 0.26(0.00) | -4.75(0.00) | 2.17(0.00) | -4.49(0.00) |
| 168SER_ARG | -15.78(0.00) | -29.88(0.00) | -86.84(0.00) | 105.70(0.00) | -4.76(0.00) | -116.72(0.00) | 100.94(0.00) |
| 17GLY_ARG | -14.61(0.00) | 9.72(0.00) | -186.17(0.00) | 166.35(0.00) | -4.51(0.00) | -176.45(0.00) | 161.84(0.00) |
| 26HIS_LYS | -13.43(0.00) | -15.99(0.00) | -138.86(0.00) | 146.24(0.00) | -4.82(0.00) | -154.85(0.00) | 141.42(0.00) |
| 168SER_ASN | -11.69(0.00) | -22.93(0.00) | -26.63(0.00) | 42.65(0.00) | -4.78(0.00) | -49.56(0.00) | 37.88(0.00) |
| 20ASP_TYR | -11.00(0.00) | -31.07(0.00) | -76.74(0.00) | 101.58(0.00) | -4.77(0.00) | -107.81(0.00) | 96.81(0.00) |
| 16GLY_THR | -1.35(0.00) | -27.67(0.00) | -5.43(0.00) | 36.48(0.00) | -4.73(0.00) | -33.10(0.00) | 31.75(0.00) |
| 20ASP_HIS | -1.24(0.00) | -28.72(0.00) | -64.93(0.00) | 97.18(0.00) | -4.76(0.00) | -93.66(0.00) | 92.41(0.00) |
| 94ILE_VAL | -1.14(0.00) | -29.18(0.00) | -3.82(0.00) | 36.61(0.00) | -4.76(0.00) | -33.00(0.00) | 31.86(0.00) |
| 112ILE_ASP | -0.78(0.00) | -29.23(0.00) | 44.50(0.00) | -11.28(0.00) | -4.76(0.00) | 15.26(0.00) | -16.04(0.00) |
| 94ILE_GLU | -0.38(0.00) | -29.05(0.00) | 23.85(0.00) | 9.56(0.00) | -4.75(0.00) | -5.19(0.00) | 4.81(0.00) |

**Supplementary TableS5** Assessment of combined virtual mutation of NadDsg by MaxFlow.

| **structure_name** | **ΔG total (kcal/mol)** | **ΔE vdw (kcal/mol)** | **ΔE ele (kcal/mol)** | **ΔE polar (kcal/mol)** | **ΔE non-polar (kcal/mol)** | **ΔG gas (kcal/mol)** | **ΔG solv (kcal/mol)** |
| --- | --- | --- | --- | --- | --- | --- | --- |
| G17R, D20Y, H26K, S168K | -87.21 | 0.50 | -507.99 | 424.82 | -4.55 | -507.48 | 420.27 |
| G17R, D20Y, H26K, S168N | -65.78 | 6.07 | -383.74 | 316.62 | -4.72 | -377.68 | 311.90 |
| G17R, D20Y, H26K, S168R | -43.39 | -12.75 | -389.87 | 364.08 | -4.84 | -402.62 | 359.24 |
